# Prognostic Value of Functional Parameters of ^18^F-FDG-PET Images in Patients with Primary Renal/Adrenal Lymphoma

**DOI:** 10.1155/2019/2641627

**Published:** 2019-07-25

**Authors:** Manni Wang, Hui Xu, Liu Xiao, Wenpeng Song, Sha Zhu, Xuelei Ma

**Affiliations:** ^1^Department of Biotherapy, State Key Laboratory of Biotherapy, Cancer Center, West China Hospital, Sichuan University, Chengdu 610041, China; ^2^Department of Radiology, West China Hospital, Sichuan University, Chengdu 610041, China; ^3^Department of Nuclear Medicine, West China Hospital, Sichuan University, Chengdu 610041, China

## Abstract

**Objectives:**

The aim of this study is to explore the textural features that may identify the morphological changes in the lymphoma region and predict the prognosis of patients with primary renal lymphoma (PRL) and primary adrenal lymphoma (PAL).

**Methods:**

This retrospective study comprised nineteen non-Hodgkin's lymphoma (NHL) patients undergoing ^18^F-FDG-PET/CT at West China Hospital from December 2013 to May 2017. ^18^F-FDG-PET images were reviewed independently by two board certificated radiologists of nuclear medicine, and the texture features were extracted from LifeX packages. The prognostic value of PET FDG-uptake parameters, patients' baseline characteristics, and textural parameters were analyzed using Kaplan–Meier analysis. Cox regression analysis was used to identify the independent prognostic factors among the imaging and clinical features.

**Results:**

The overall survival of included patients was 18.84 ± 13.40 (mean ± SD) months. Univariate Cox analyses found that the tumor stage, GLCM (gray-level co-occurrence matrix) entropy, GLZLM_GLNU (gray-level nonuniformity), and GLZLM_ZLNU (zone length nonuniformity), values were significant predictors for OS. Among them, GLRLM_RLNU ≥216.6 demonstrated association with worse OS at multivariate analysis (HR 9.016, 95% CI 1.041–78.112, *p*=0.046).

**Conclusions:**

The texture analysis of ^18^F-FDG-PET images could potentially serve as a noninvasive strategy to predict the overall survival of patients with PRL and PAL.

## 1. Introduction

Renal involvement has been reported as a common situation in patients diagnosed with non-Hodgkin's lymphoma (NHL) [1]. Primary renal lymphoma (PRL), though less common than secondary renal lymphomas [2], is an important and lethal type of extranodal lymphomas [3]. Unlike secondary renal masses which arise from invasion of an adjacent lymphomatous mass, PRL usually originates from renal parenchyma and is highly aggressive due to its rapid dissemination [4]. Currently reported symptoms include pain, anorexia, vomiting, fever, hypertension, palpable renal masses, hematuria, and acute kidney injury [4, 5]. It has been reported that the median survival of PRL is usually less than 1 year [4, 5], which may be attributed to the recurrence and neutropenia-related infection [6]. Surgical resection, chemotherapy, and consolidation radiotherapy can improve the disease-free and overall survival [7]. Likewise, primary adrenal lymphoma (PAL) is also a rare form of cancer, of which fewer than 200 cases have been reported [8], and the prognosis is generally poor [9]. Despite the rare existence of PAL and PRL, it is important to shed light on the potential factors related to their prognosis to stratify treatment among individual patients.

^18^F-Flourodeoxyglucose (^18^F-FDG) positron emission tomography (PET)/computed tomography (CT), which provides functional as well as anatomic imaging information, has long been recognized as a powerful imaging technique for the clinical evaluation and diagnosis of lymphoma [10]. However, the detection of renal lymphoma with ^18^F-FDG-PET remains challenging since the kidney does not carry lymphoid tissues and FDG is able to distribute into normal kidney tissues. On the contrary, limited literatures have reported the utility of ^18^F-FDG-PET in detecting the metabolic activity of PAL [11, 12]. The PET/CT scan has clinically been utilized to distinguish between PAL and secondary adrenal lymphomas [8] and to follow-up on treatment responses [13]. Of all the parameters of ^18^F-FDG-PET images, the maximum standardized uptake value (SUVmax) is one of the most commonly used indexes to predict patients' prognosis and their therapeutic responses [14]. In addition, the metabolic tumor volume (MTV) and total lesion glycolysis (TLG) are suggested to provide more accurate prediction on the tumor burden, tumor behavior, as well as treatment response [15].

Recently, a novel technique has been proposed to help predict the clinical outcome and treatment response of various types of tumor [16, 17]. The texture analysis, based on the theory that images containing a complex visual pattern, allows the mathematic detection of the subtle spatial arrangement of the gray level among image pixels [18, 19]. Tumor uptake of ^18^F-FDG varies due to the necrosis, cell proliferation, microvessel density, and hypoxia within tumors [20–22]. There has been considerable interest in examining the correlation of textural features and ^18^F-FDG-PET parameters of PET images with survival outcomes [23–28]. Given the ability of texture analysis to detect subtle pathologic changes in an ^18^F-FDG-PET image, we herein compared the texture features of PET images of 19 patients with PNL or PAL. The aim of this study was to explore textural features that may potentially identify the morphological changes of lymphoma regions and predict prognosis of PNL and PAL, which to the best of our knowledge, is the first of its kind.

## 2. Methods

### 2.1. Patients

This retrospective study was approved by the institutional ethics committee of West China Hospital, Sichuan University, and no written informed consent was required. Patients with pathologically confirmed PRL or PAL who underwent ^18^F-FDG-PET/CT scans at West China Hospital between December 2013 and May 2017 were enrolled in this study. Patients were considered eligible based on the following criteria: (1) pathologically confirmed primary renal lymphoma or primary adrenal lymphoma via either biopsy or surgery; (2) the primary tumor with visible abnormal ^18^F-FDG uptake; and (3) ^18^FDG-PET/CT scans performed to characterize a kidney lesion or adrenal gland lesion. Patients were excluded if follow-ups were less than 12 months due to other causes of death. All patients were followed for at least 12 months according to our institutional protocol. Local recurrence and distant metastasis were confirmed with imaging techniques and, if possible, histopathologic examination by either biopsy or surgical excision. Overall survival (OS) was defined as the period between the date of diagnosis and death. Patients who did not experience recurrence or metastasis at the end of follow-ups were recorded as censored.

### 2.2. Imaging Protocols

Whole-body PET/CT examinations were performed before the beginning of any treatment, using a combined Gemini GXL PET/CT scanner with a 16-slice CT component (Philips Medical System, Cleveland, Ohio, USA). Original images were retrieved from the picture archiving and communication system (PACS). We uniformly used 5.0 mm slice CT images, and image processing was mainly in cross sections. All patients were instructed to fast for 6 hours (no oral or intravenous fluids containing sugar or dextrose) before examinations. Immediately before ^18^F-FDG injection, the blood glucose level was measured and PET/CT scans would be rescheduled if it was higher than 150 mg/dl. Image acquisition started at 60 ± 5 minutes after intravenous injection of ^18^F-FDG (3.7 MBq/kg). Emission data were acquired for 2 minutes per bed position. CT from the head to the feet was performed before PET which covered an identical area with CT. Image registration and fusion of PET and CT scans were carried out with Syntegra software, Philips Corp., Amsterdam. The autorandom correction and autoscatter correction were applied, and the corrected images were reconstructed with 2 *∗*2 *∗* 2 mm^3^ voxels using line of response (LOR), without postreconstruction filtering.

### 2.3. Image Analysis

The focal ^18^F-FDG uptake at the primary tumor was reviewed independently by two board certificated radiologists specialized in nuclear medicine, blinded to the patient history. Any disagreement was resolved by a third nuclear medicine radiologist. To exclude adjacent physiological ^18^F-FDG-avid structures and ensure that VOI (volume of interest) was restricted to the baseline tumor, the VOI was manually drawn with consensus by three nuclear medicine-certified radiologists together. To avoid the interference of the lower image matrix resolution, the images were excluded if VOI did not reach the minimum number of 64 voxels. The SUVmax and SUVmean were defined as the maximum and mean radioactivity concentration of images enclosed by the VOI divided by the whole-body concentration of the injected radioactivity. SUVmax, SUVmean, and MTV values were then measured automatically using commercial software (Advantage Windows Workstation; GE Healthcare, Milwaukee, WI). TLG was calculated as SUVmean *∗* MTV.

### 2.4. Textual Analysis

The texture analysis was performed inside the VOI retrieved from PET images. Features of the primary tumor were extracted using the LifeX package (http://www.lifexsoft.org) [29]. Given that not all of the texture parameters were helpful for the differential diagnosis [30, 31], tumor uptake heterogeneity was analyzed only with robust heterogeneity parameters according to previous reports [25, 32]. A set of 37 texture indices included (1) five histogram indices: HISTO_Skewness, HISTO_Kurtosis, HISTO_Entropy_log10, HISTO_Entropy_log12, and HISTO_Energy; (2) seven gray-level co-occurrence matrix (GLCM) features: GLCM_Homogeneity, GLCM_Energy, GLCM_Contrast, GLCM_Correlation, GLCM_Entropy_log10, GLCM_Entropy_log2, and GLCM_Dissimilarity; (3) eleven gray-level run-length matrix (GLRLM) features: GLRLM_SRE, GLRLM_LRE, GLRLM_LGRE, GLRLM_HGRE, GLRLM_SRLGE, GLRLM_SRHGE, GLRLM_LRLGE, GLRLM_LRHGE, GLRLM_GLNU, GLRLM_RLNU, and GLRLM_RP; (4) three gray-level gradient matrix (NGLDM) features: NGLDM_Coarseness, NGLDM_Contrast, and NGLDM_Busyness; (5) and eleven gray-level run-length matrix (GLZLM) features: GLZLM_SZE, GLZLM_LZE, GLZLM_LGZE, GLZLM_HGZE, GLZLM_SZLGE, GLZLM_SZHGE, GLZLM_LZLGE, GLZLM_LZHGE, GLZLM_GLNU, GLZLM_ZLNU, and GLZLM_ZP. The FDG uptake intensity data was rescaled using 64 discrete values to reduce the image noise.

### 2.5. Statistical Analysis

The receiver-operating-characteristic (ROC) analyses were performed, and the area under the ROC curves (AUCs) was calculated to identify the optimal cutoff values for each texture parameter. All patients were then dichotomized into high- and low-value groups using cutoff values calculated with the Youden index [33]. Survival curves were drawn with the Kaplan–Meier method, and the log-rank test was performed to testify the significance of difference between each pair of survival curves. Cox regression models were used to calculate hazard ratios (HRs) and to determine the effects of clinicopathological characteristics and selected texture parameters on OS. We first performed univariate analyses on a series of variables, followed by multivariate analyses on selected variables with significant association in the univariate analysis. The *p* value <0.05 was considered as statistically significant, and all *p* values presented were two-sided. All statistical analyses were performed using IBM SPSS Statistics for Windows (version 19.0, IBM Corp. Armonk, NY).

## 3. Results

### 3.1. Baseline Characteristics

Nineteen patients, 12 males and 7 females, were included in this study. The median age was 52.16 ± 15.06 years. Nine of them were alive at the end of follow-ups (December, 2017). The overall survival was 18.84 ± 13.40 (mean ± SD) months. All 19 patients had visible tumors on ^18^F-FDG-PET at the time of diagnosis. The ^18^F-FDG-SUVmax values ranged from 1.50 to 24.28, and the ^18^F-FDG-SUV mean ranged from 1.0 to 25.6. The ^18^F-FDG-MTV ranged from 1.0 to 869.2 cm^3^, and the corresponding ^18^F-FDG-TLG ranged from 3.6 to 7840.2 cm^3^.

A total number of 9 (47.4%) non-Hodgkin's lymphomas developed in the kidney. 8 (42.1%) occurred in the adrenal gland, and 2 (10.5%) cases involved both organs. Based on the Ann Arbor staging system, 10.5% of patients were classified as stage I, while stage II, III, and IV patients accounted for 5.3%, 26.3%, and 57.9%, respectively. Furthermore, according to the origin of tumor cells, 13 (68.4%) lymphomas were classified as B-cell lymphoma, 5 (26.3%) as NK-cell lymphoma, and only 1 (5.3%) case as T-cell lymphoma (Table 1).

### 3.2. ROC Analyses and Cutoff Values for Parameters

The receiver-operating curve (ROC) was used to identify the optimal cutoff value of a parameter. The parameter was more likely to accurately identify a positive instance (worse prognosis) when the AUC value was high (*p* < 0.05). The ten texture parameters with the highest AUC values were considered potentially discriminative and, together with four ^18^F-FDG-PET parameters, were included in further analyses. By analyzing the specificity and sensitivity of each parameter, we took the optimal cutoff values of SUVmax, SUVmean, MTV, and TLG as 7.37, 7.00, 88.80, and 13.05, respectively. The AUC values of ^18^F-FDG-PET parameters for predicting overall survival were 0.578 (*p*=0.568) for SUVmax, 0.589 (*p*=0.514) for SUVmean, and 0.722 (*p*=0.102) for MTV, and 0.733 (*p*=0.086) for TLG. The ability of each image-based parameter to predict OS at the optimal threshold is summarized in Table 2.

As for the ten texture parameters enrolled in this study, all exhibited statistical significance in prognosis prediction (*p* < 0.05). The AUC values were 0.800 for HISTO_Entropy, 0.867 for GLCM_Entropy, 0.867 for GLCM_Correlation, 0.794 for GLRLM_HGRE (high gray-level run emphasis), 0.778 for GLRLM_SRHGE (short-run high gray-level emphasis), 0.800 for GLRLM_LRLGE (long-run high gray-level emphasis), 0.878 for GLRLM_RLNU (run-length nonuniformity), 0.778 for GLZLM_HGZE, (high gray-level zone emphasis), 0.844 for GLZLM_GLNU (gray-level nonuniformity), and 0.856 for GLZLM_ZLNU (zone length nonuniformity). The corresponding optimal cutoff values are presented in Table 2. For further analyses, patients were then dichotomized into two categories: less than and no less than the cutoff values.

### 3.3. Survival Prediction

**Figure 1:**
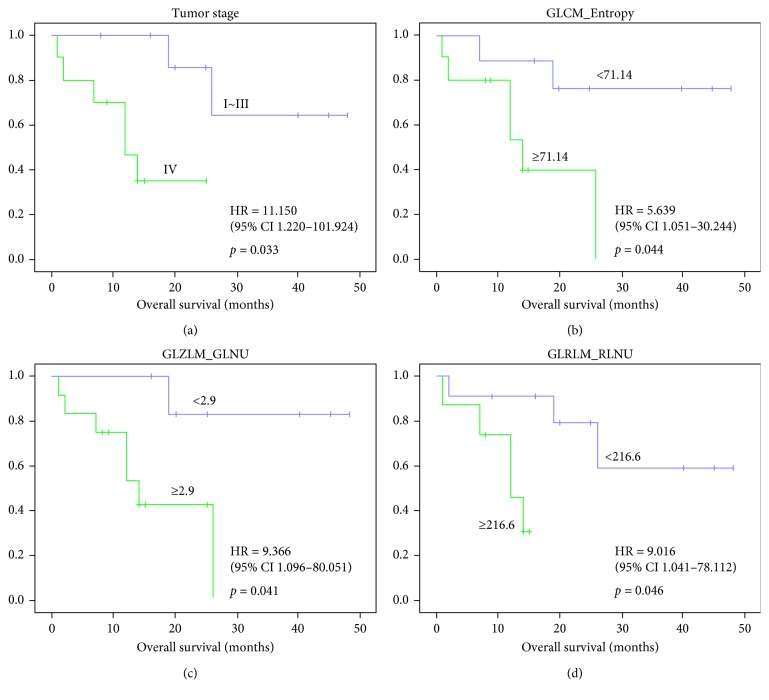
Kaplan–Meier survival curves of the overall survival of patients with PAL and PNL. Stages: (a) GLCM_Entropy, (b) GLZLM_GLNU, and (c) GLRLM_RLNU. (d) The significant differences are demonstrated in each parameter.

The median OS for all patients was 15 (range, 9–25) months. At the end of the follow-up, nine patients eventually died and ten patients were alive. The Cox regression analyses, performed to assess the impact of each parameter on survival outcomes, are presented in Table 3. The clinicopathological characteristics including age, gender, location, stage, and tumor cell origin were all enrolled in the univariate analysis, while only the Ann Arbor stage revealed a significant association with OS. Patients of stage IV renal/adrenal lymphoma displayed worse survival outcome compared with those of stage I–III (HR 11.150, 95% CI 1.220–101.924, *p*=0.033). The Kaplan–Meier survival curves for the overall survival stratified by the tumor stage, GLCM_Entropy, GLZLM_GLNU, and GLRLM_RLNU are shown in Figure 1.

The four ^18^F-FDG-PET parameters, SUVmax, SUVmean, MTV, and TLG, were also enrolled in the univariate Cox analysis. Although a longer mean OS was observed in the low MTV group compared with the high MTV group (21.13 vs. 10.25 months), the difference failed to demonstrate a statistical significance (*p*=0.052). The univariate analysis suggested the association between texture parameters and OS of patients with PAL and PRL. High values of GLRLM_RLNU were significantly correlated with poorer OS (25.00 vs. 10.38 months, *p*=0.046). Moreover, compared with that of the high GLZLM_GLNU group, the OS was markedly improved in patients with low GLZLM_GLNU (30.43 months vs. 12.08 months, *p* < 0.001). The low GLCM_Entropy value served as another potential predictor for favorable prognosis (27.22 months vs. 11.30 months, *p*=0.044).

**Figure 2:**
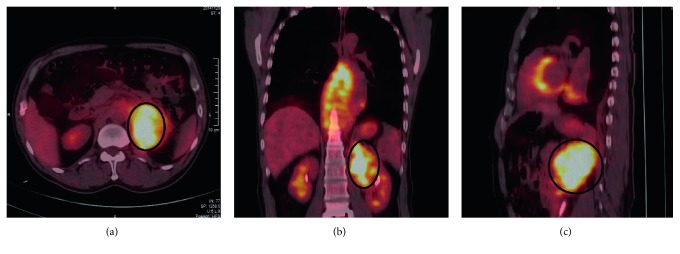
^18^F-FDG-PET/CT images of a 63-year-old male patient with non-Hodgkin's lymphoma on the left adrenal gland. He was alive at the end of our follow-ups with no disease progression. The green lines represent the borders of the VOI. The GLRLM_RLNU value of his PET images was 55.8 (lower than our cutoff value 216.6)

The multivariate analysis was accordingly performed to identify any independent prognostic factors for PAL and PRL. Potential factors enrolled in the multivariate analysis included the tumor stage, GLRLM_RLNU, GLZLM_GLNU, and GLCM_Entropy values. Among them, the GLRLM_RLNU ≥216.6 showed a significant association with patients' survival outcome in multivariate analysis (HR 9.016, 95% CI 1.041–78.112, *p*=0.046). However, none of the other texture parameters appeared to be independent predictors for the prognosis of the patients with primary renal/adrenal lymphoma. The representative ^18^F-FDG-PET/CT images of patients with relatively long overall survival are shown in Figure 2.

## 4. Discussion

The prognosis prediction before treatment can be of great value to modulate treatment strategies and therefore optimize therapeutic results. The aim of this study was to explore textural features that may potentially identify the morphological changes of the lymphoma region and predict prognosis of PNL and PAL. To the best of our knowledge, this is the first study to examine the correlation of baseline ^18^F-FDG-PET image-based parameters including the uptake indices (SUVmax, SUVmean, MTV, and TLG) and 37 texture parameters with the survival outcomes of PRL and PAL.

The results from our study suggest a potential association between the PET image-derived parameters with OS in patients with renal and adrenal NHL. The tumor stage, GLRLM_RLNU, GLZLM_GLNU, and GLCM_Entropy values, were shown as significant predictors for OS at the univariate analysis. After adjusting for the above variables, the high level of GLRLM_RLNU was shown to be independently associated with poor survival. These results suggested that PET texture analysis could potentially be utilized as an independent indicator for the prognosis of patients with PRL and PRL. In terms of survival prediction, image-derived texture features outperformed ^18^F-FDG-uptake indices and common clinical predictors including Ann Arbor staging. Our findings are in line with a recent study that revealed no significant interaction between pretreatment FDG-uptake values and the survival of patients with HL and aggressive NHL [34]. On the contrary, one study reported that MTV, but not other uptake parameters, was an independent prognostic factor for patients with esophageal cancer [35]. In our study, although the MTV lacks statistical significance for the correlation with OS (*p*=0.052), other parameters including SUVmax, SUVmean, and TLG demonstrated higher *p* values. In another study, MTV was not an independent factor for prognosis in patients with esophageal cancer [36]. All these differences may be attributed to the different study populations.

Previous studies reporting PRL and PAL cases have demonstrated the rare existence of PRL and PAL [5, 7, 13]. Due to the absence of lymphatic tissues in the kidney, PRL has long been questioned about its existence. Several theories on the origin of PRL have been proposed. One possible mechanism appears to involve the chronic inflammation of the kidney promoting the invasion of lymphoid cells, followed by the oncogenic transformation of those cells in situ. Another potential mechanism focuses on that lymphatic channels surrounding the renal capsule from which renal lymphomas originate and infiltrate the renal parenchyma [37]. Currently, the Ann Arbor method [38], first introduced in 1971 and revised in 1989 to incorporate the “Cotswolds modifications,” is the most widely accepted staging system for both Hodgkin lymphoma and non-Hodgkin lymphoma [39, 40]. It divides Hodgkin lymphoma (HL) patients into four stages with subclassifications of A or B based on the presence of disease-related symptoms such as fevers to greater than 101°F (38.3°C), weight loss, and night sweats [41]. Considering the low survival rate of PRL and PAL patients [5, 42], prediction of prognosis could be of great significance to optimize the clinical management of PRL patients.

Although the texture analysis has recently been identified as a noninvasive approach that simultaneously provides information at the image acquisition, very limited studies have highlighted its role in lymphoma using radiological images such as PET, CT (computed tomography), and MRI (magnetic resonance imaging). Textural features of pretreatment FDG-PET images are able to predict cancer recurrence and patient survival [43–45]. On the contrary, PET image-derived parameters such as SUVmax are also commonly utilized in radiomic studies [46]. Patients' clinicopathological characteristics should be concomitantly analyzed, given that they might also have an influence in image variables and textural features [47]. Numerous efforts have been undertaken in molecular imaging with ^18^F-FDG-PET which helps stratify diagnosis, staging, and response assessment in lymphoma patients [48, 49]. It is suggested that a baseline pretreatment scan should be undertaken to allow meaningful comparison before and after treatment [50, 51]. Clinically, the ^18^F-FDG-PET is not only used in predicting treatment outcome of patients with solid tumors but also widely used in FDG-avid lymphoma with indications depending on specific diagnosis and presentation. On some occasions such as organ involvement, the ^18^F-FDG-PET/CT displays superior accuracy to the CT scan alone, where PET/CT showed sensitivity of 88% and specificity of 100% versus sensitivity of 50% and specificity of 90% with contrast-enhanced CT alone [52].

The major limitation of our study is the relatively small sample size. The primary adrenal/renal lymphoma is rare and less commonly seen than the secondary adrenal/renal lymphoma, which results in the limited number of patients we and other studies could reach [34]. Another limitation is that this study only included NHL patients, while the mixed nature of different lymphoma subtypes (HL and UHL) could possibly influence the results. However, a previous study suggested that there was no significant difference in CT texture analysis indices between HL and NHL. It is therefore interesting to explore whether the differences exist in PET images. Future studies with larger sample size are warranted to evaluate the prognostic value of PET image-texture analysis in more lymphoma types.

## 5. Conclusions

Despite the rarity of their existence, it is intriguing to speculate the prognostic factors of the PRL and PAL. Our findings demonstrate that the radiomic parameters derived from baseline PET images, such as GLRLM_RLNU, GLZLM_GLNU, and GLCM_Entropy, were predictive of overall survival in patients. Therefore, the texture analysis of ^18^F-FDG-PET images could potentially serve as a noninvasive strategy to predict the overall survival of patients with PRL and PAL. Further studies with a larger sample size are warranted to validate this predictive model.

## Figures and Tables

**Table 1:** Baseline characteristics of patients (*n* = 19).

Characteristics		No. of patients (%)
Age (year)	<50	8 (42.1)
	≥50	11 (57.9)
	Mean ± SD	52.16 ± 15.06

Sex	Male	12 (63.2)
	Female	7 (36.8)

Tumor location	Kidney	9 (47.4)
	Adrenal gland	8 (42.1)
	Kidney + adrenal gland	2 (10.5)

Ann Arbor stage	I	2 (10.5)
	II	1 (5.3)
	III	5 (26.3)
	IV	11 (57.9)

Subtype	B-cell lymphoma	13 (68.4)
	T-cell lymphoma	1 (5.3)
	NK-cell lymphoma	5 (26.3)

**Table 2:** Area under ROC curves (AUCs) and optimal thresholds of ^18^F-FDG-PET and texture parameters to predict OS.

Parameters	AUC	95% confidence intervals	*p* value	Optimal cutoff value
^*18*^ *F-FDG-PET*				
SUVmax	0.578	0.308–0.847	0.568	7.37
SUVmean	0.589	0.318–0.86	0.514	7.00
MTV	0.722	0.487–0.957	0.102	88.80
TLG	0.733	0.506–0.961	0.086	13.05

*Texture analysis*				
GLCM_Correlation	0.867	0.703–1.000	0.007	0.66
GLRLM_HGRE	0.794	0.576–1.000	0.030	171.95
GLRLM_SRHGE	0.778	0.553–1.000	0.041	163.20
GLRLM_LRHGE	0.800	0.585–1.000	0.027	215.80
GLRLM_RLNU	0.878	0.719–1.000	0.006	216.60
GLZLM_HGZE	0.778	0.555–1.000	0.041	205.05
GLZLM_GLNU	0.844	0.661–1.000	0.011	2.90
GLZLM_ZLNU	0.856	0.685–1.000	0.009	9.75
HISTO_Entropy	0.800	0.582–1.000	0.027	12.60
GLCM_Entropy	0.867	0.695–1.000	0.007	71.14

**Table 3:** Univariate and multivariate Cox regression analyses of factors associated with OS.

Variables		Median OS (month)	Univariate analysis		Multivariate analysis	
			HR (95% CI)	*p*	HR (95% CI)	*p*
Age (year)	<50	20.63		0.745		
	≥50	17.55				
Gender	Male	17.00		0.347		
	Female	22.00				
Location	Kidney	14.00		0.432		
	Adrenal gland	17.50				
	Kidney + adrenal gland	20.00				
Ann Arbor stage	I∼III	27.44	11.150 (1.220–101.924)	0.033^*∗*^		
	IV	11.10				
Subtype/origin	B cell	21.62		0.244		
	T cell	2.00				
	NK cell	15.00				

^*18*^ *F-FDG-PET parameters*						
SUVmax	<7.37	25.20		0.264		
	≥7.37	16.57				
SUVmean	<7.00	23.25		0.575		
	≥7.00	15.64				
MTV	<88.80	21.13	5.044 (0.983–25.882)	0.052		
	≥88.80	10.25				
TLG	<13.05	31.00		0.065		
	≥13.05	15.60				

*Texture features*						
GLCM_Correlation	<0.695	22.38	5.089 (0.911–28.421)	0.064		
	≥0.695	11.17				
GLRLM_HGRE	<171.95	28.13		0.076		
	≥171.95	12.09				
GLRLM_SRHGE	<163.2	28.13		0.076		
	≥163.2	12.09				
GLRLM_LRHGE	<215.8	28.13		0.076		
	≥215.8	12.09				
GLRLM_RLNU	<216.6	25.00	9.016 (1.041–78.112)	0.046^*∗*^	9.016 (1.041–78.112)	0.046^*∗*^
	≥216.6	10.38				
GLZLM_HGZE	<205.05	28.13		0.076		
	≥205.05	12.09				
GLZLM_GLNU	<2.9	30.43	9.366 (1.096–80.051)	0.041^*∗*^		
	≥2.9	12.08				
GLZLM_ZLNU	<9.75	28.13		0.076		
	≥9.75	12.09				
HISTO_Entropy	<12.6	28.13		0.076		
	≥12.6	12.09				
GLCM_Entropy	<71.14	27.22	5.639 (1.051–30.244)	0.044^*∗*^		
	≥71.14	11.30				

^∗^ *p* < 0.05.

## Data Availability

The data used to support the findings of this study are available from the corresponding author upon request.
